# Novel approach to the maxillary sinusitis after sinus graft

**DOI:** 10.1186/s40902-017-0115-3

**Published:** 2017-06-25

**Authors:** Sung ok Hong, Gyu-Jo Shim, Yong-Dae Kwon

**Affiliations:** 1 Department of Dentistry (Oral and Maxillofacial Surgery), Catholic Kwandong University, School of Medicine, International St. Mary’s Hospital, Incheon, Republic of Korea; 2 Department of Oral and Maxillofacial Surgery, School of Dentistry, Kyung Hee University, Seoul, Republic of Korea; 3 Department of Oral and Maxillofacial Surgery, Graduate School of Dentistry, Kyung Hee University, Seoul, Republic of Korea

**Keywords:** Rhinosinusitis, Maxillary sinusitis, Sinusitis treatment

## Abstract

**Background:**

Postoperative infection occurs when bone graft material is dislodged into the maxillary sinus cavity and most of the patients are often uncomfortable with the drainage and irrigation procedures to eradicate the infection. In this case report, we share a technique in treating patients with such condition.

**Material and methods:**

A 47-year-old patient was referred after sinus elevation using the crestal socket osteotome, bone graft, and implant insertion at a local clinic. Clinical and radiographic findings confirmed the diagnosis of right maxillary sinusitis. A surgical and medical treatment regimen was applied.

**Results:**

By using this technique for irrigation, we were able to achieve successful results, and the patient was satisfied due to less discomfort during the irrigation process.

**Conclusion:**

This method is a patient-friendly technique for sinus irrigation. It is not only limited to sinus grafted patients, but also maxillary sinusitis patients in any other type of odontogenic infection.

## Background

Rhinosinusitis can be divided into four classifications regarding the sign, symptom, and course of disease- acute, subacute, recurrent, and chronic [1]. Ten to 12% of maxillary sinusitis have been contributed by odontogenic etiology, and current literature has also reported dental origin to account for 30–40% of chronic maxillary rhinosinusitis [2, 3]. Rhinosinusitis occurs when the Schneidarian membrane is perforated or disrupted by infections due to dental tooth infection, maxillary trauma, bone pathologies, foreign bodies in the sinus, cysts, supernumerary teeth, implant insertion, dental extraction, orthognathic surgery, sinus membrane elevation, etc. [4].

Dental procedures such as the lateral antrostomy, which was initially described by Tatum in 1976 and published by Boyne and James in 1980, allows bone regeneration in the maxilla where residual bone height is not sufficient for implant insertion. This technique requires an antrostomy window on the lateral wall of the sinus, followed by elevation of the membrane and bone grafting. Another approach is through the crest of the alveolar ridge, otherwise called the crestal lift technique. Both techniques can provoke sinus perforation and displacement of graft materials into the sinus, thus leading to sinusitis. In this study, we would like to share one technique in treating patients with such conditions. This method is not only limited to sinus grafted patients, but also maxillary sinusitis patients due to any other type of odontogenic infection.

## Case presentation

### Methods

**Fig. 1:**
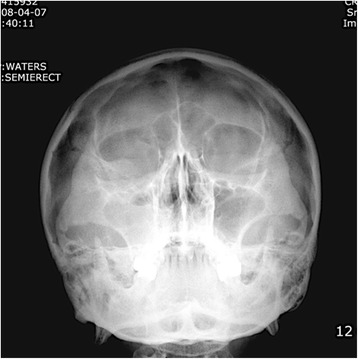
Waters’ view shows haziness of the right maxillary sinus which coincides with the diagnosis of acute sinusitis

A 47-year-old patient was referred to our department with the chief complaint of foul odor, tenderness on the right sinus, and headache. Sinus elevation using the crestal socket osteotome, bone graft, and implant insertion had been initiated at a local clinic 10 days previously. Further clinical examinations showed gingival redness and swelling of the right buccal gingiva, pus discharge from the right nose, and lymphadenopathy of the right. A Waters’ view plain-film radiograph showed haziness of the right maxilla confirming sinusitis of the right maxilla (Fig. 1).

**Fig. 2:**
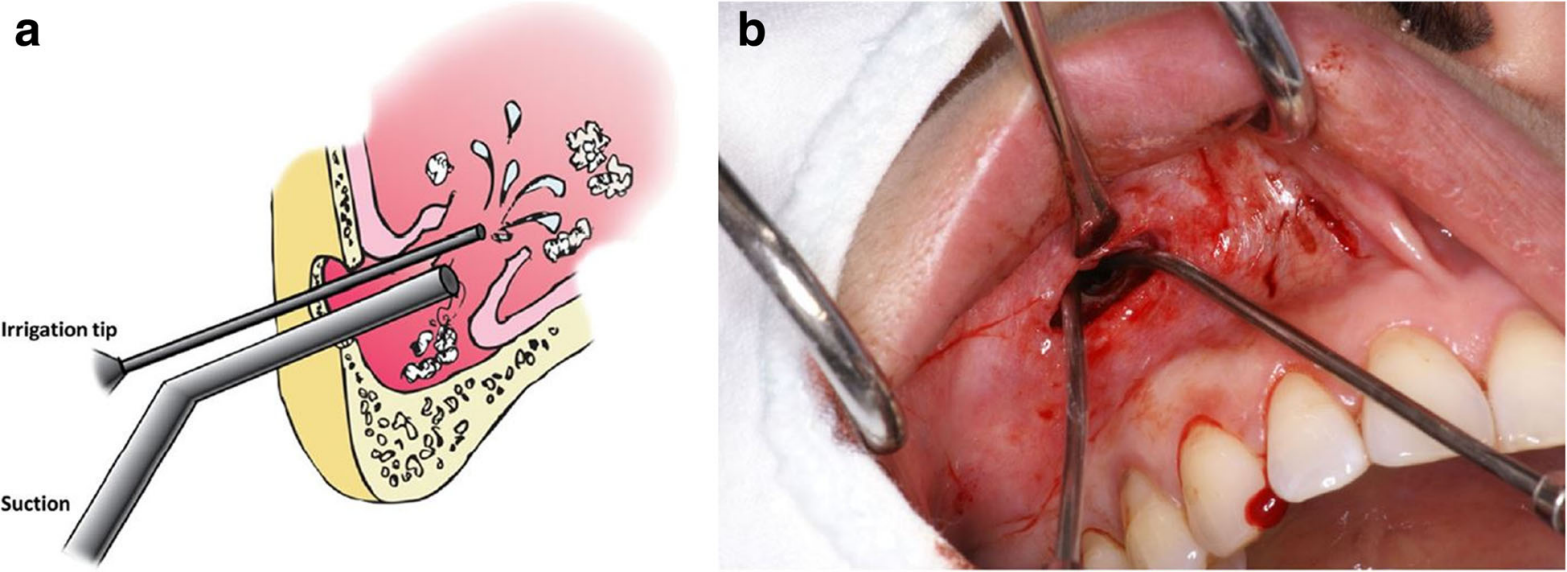
**a** Illustration depicting how the infected graft materials are being irrigated and suctioned through an anthrostomy window of the anterior wall of the sinus. **b** Irrigation and suction through an anthrostomy window of the anterior wall of the sinus

**Fig. 3:**
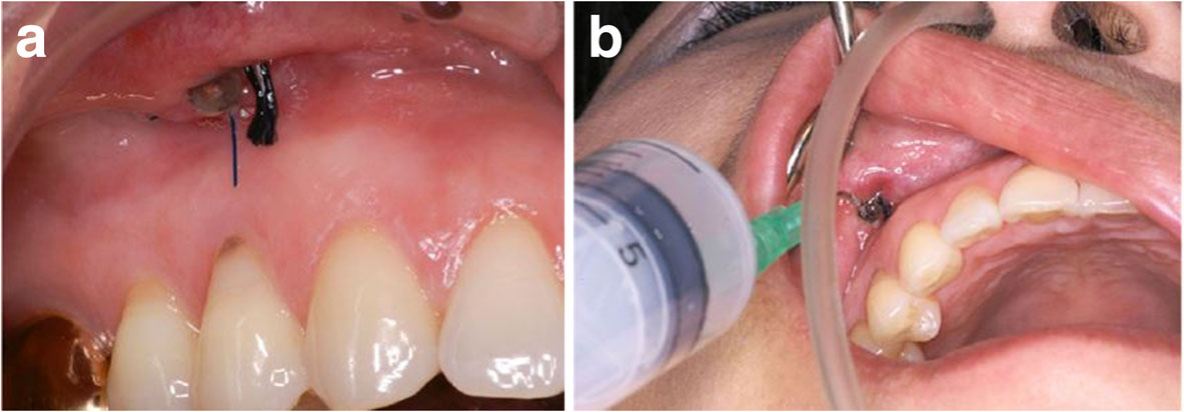
**a** Copious irrigation with saline through a tube drain that has been inserted intraorally through the right anthrostomy window is being performed. **b** A flexible silastic suction is placed in the nostril to prevent saline from flowing out of the nose and mouth

Explantation of the infected implant and sinus irrigation was initiated. Sinus irrigation was conducted by lateral antrostomy through the canine fossa. The canine fossa is the thinnest area of the anterior wall and is easily accessed. Through the window, pus was aspirated using a syringe, and cultured. The remaining infected bone graft materials were removed by inserting a suction and irrigation tip into the window and lightly irrigating with saline solution (Fig. 2a, b). A tube drain was inserted and then sutured to the gingiva. A flexible silastic suction tip was inserted into the right nostril towards the ostio-meatal complex. Further copious irrigation of the sinus was initiated through the silastic drain in the oral cavity (Fig. 3a, b).

Four consecutive irrigation sessions 4 days apart were executed, and the drain was removed on the last day of irrigation. Amoxicillin (500 mg), pseudoephedrine hydrochloride (60 mg), carbocisteine (750 mg), and NSAIDs were given three times daily for a total of 21 days.

### Results

**Fig. 4:**
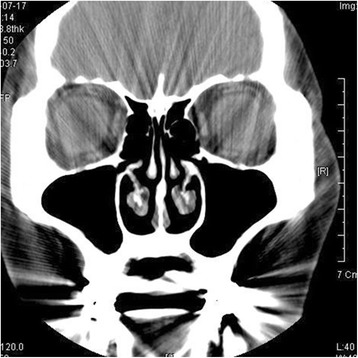
Computed tomography (CT) view at 3.5 months follow-up. Radiolucency is shown on both sinuses and all clinical symptoms of right side sinusitis have been relieved

After removal of the infected bone graft materials and implant, the patient had immediately relieved symptoms of headache. After four irrigation sessions, discomfort on the right buccal area had diminished, and pus accumulation was not observed. The microbiological culture of the maxillary sinus on the first day of treatment revealed the presence of α-hemolytic *Streptoccus viridans*. A follow-up computed tomography (CT) at 3.5 months displayed radiolucency in the infected right maxillary sinus, and was conclusive of recovery (Fig. 4).

### Discussion

Odontogenic and non-odontogenic sinusitis differ in cause, pathophysiology, and microbiology. Therefore, specifically identifying the cause is the first step for a successful outcome. Elimination of the source of infection is the essential step to relieve symptoms and prevent recurrence of sinusitis. Odontogenic sinusitis can be treated medically and/or surgically. Medical treatment regimens are based on antibiotics, determined through bacterial culture, and decongestants. Administrating antibiotics is a crucial step in managing odontogenic sinusitis. One study found α-hemolytic *Streptoccus viridans*, microaerophilic streptococci, and *Stapylococcus aureus* to be the most common aerobic bacteria, and anaerobic gram-negative bacilli, *Peptostreptococcus* spp, and *Fusobacterium* spp to be the most common anaerobic bacteria [3]. These findings are interesting since most common non-odontogenic origin microbes are *Streptococcus pneumonia*, *Haemophilus influenza*, and *Moraxella catarrhalis* [5]. Therefore, antibiotic selection should be carefully contemplated following a pus culture such as in our case. Quick and accurate measures must be taken sine oral antibiotics are only effective against oral flora and sinus pathogens for only 21 to 28 days [6].

Surgical regimens may vary depending on the etiology of rhinosinusitis. Lechien et al [4] did a review that studied the proportion owing to odontogenic chronic maxillary sinusitis. Iatrogenic, marginal periodontitis, apical periodontitis, apical granuloma, odontogenic cyst, odontoma, ectopic tooth, peri-implantitis were all reviewed. Iatrogenic etiology accounted for most of the cases being 65.7%.

The classic Caldwell luc approach was commonly used, despite its morbidity and 9–15% recurrence rate, for chronic odontogenic sinusitis [7]. Therefore less invasive endoscopic sinus surgery (ESS) techniques have been promoted because it is safer, quicker, has less impact on the sinus mucus clearance, advocates less bleeding, and allows for a shorter hospital stay [4]. But, it has limitation of exposure to the anterior maxillary anterior wall and lacrimal recess, and access to the anterior wall [8]. Some recent literature have proposed puncturing the canine fossa in combination of ESS [7], while some recommend using a dilated balloon [9]. But, most of the recent methods require the practitioner to have access to an endoscope or they are not adequate for odontogenic cases.

## Conclusions

In many cases, obtaining an endosope is expensive and it seems impractical investing just for the use of such complications. Our technique provides office-based surgery and irrigation with minimal discomfort for the patient. When this procedure is compared with the treatment of other maxillary sinusitis such as Caldwell-Luc surgery, ESS, it has some advantages such as lower complication rates, less blood loss and operation time, and lower cost. Also, because the surgical procedure is not complicated, local clinics have an advantage in that it can be used under local anesthesia without difficulty. But, unlike other treatments, it is a blind technique that irrigation must be performed sufficiently with accurate anatomical knowledge of the maxillary sinus. This method is a patient friendly technique for sinus irrigation and highly recommended.
